# Context factors in general practitioner - patient encounters and their impact on assessing communication skills - an exploratory study

**DOI:** 10.1186/1471-2296-14-65

**Published:** 2013-05-22

**Authors:** Geurt Essers, Anneke Kramer, Boukje Andriesse, Chris van Weel, Cees van der Vleuten, Sandra van Dulmen

**Affiliations:** 1 Department of Primary & Community Care, Radboud University Nijmegen Medical Centre, Nijmegen, The Netherlands; 2 Bureau BRIES, Independent consultancy for primary care, Nieuwkoop, The Netherlands; 3 Australian Primary Health Care Research Institute, Australian National University, Canberra, Australia; 4 Department of Educational Development and Research, Maastricht University, Maastricht, The Netherlands; 5 Northumbria University, Newcastle upon Tyne, UK; 6 Copenhagen University, Copenhagen, Denmark; 7 NIVEL (Netherlands Institute for Health Services Research), Utrecht, The Netherlands; 8 Department of Health Science, Buskerud University College, Drammen, Norway; 9 Department of Primary & Community Care, Radboud University Nijmegen Medical Centre, Geert Grooteplein 21, 6525 EP Nijmegen, The Netherlands

**Keywords:** Communication and Interviewing skills, Continuing Medical Education, Graduate Medical Education, Assessment of Learner Performance

## Abstract

**Background:**

Assessment of medical communication performance usually focuses on rating generically applicable, well-defined communication skills. However, in daily practice, communication is determined by (specific) context factors, such as acquaintance with the patient, or the presented problem. Merely valuing the presence of generic skills may not do justice to the doctor’s proficiency.

Our aim was to perform an exploratory study on how assessment of general practitioner (GP) communication performance changes if context factors are explicitly taken into account.

**Methods:**

We used a mixed method design to explore how ratings would change. A random sample of 40 everyday GP consultations was used to see if previously identified context factors could be observed again. The sample was rated twice using a widely used assessment instrument (the MAAS-Global), first in the standard way and secondly after context factors were explicitly taken into account, by using a context-specific rating protocol to assess communication performance in the workplace. In between first and second rating, the presence of context factors was established. Item score differences were calculated using paired sample t-tests.

**Results:**

In 38 out of 40 consultations, context factors prompted application of the context-specific rating protocol. Mean overall score on the 7-point MAAS-Global scale increased from 2.98 in standard to 3.66 in the context-specific rating (p < 0.00); the effect size for the total mean score was 0.84. In earlier research the minimum standard score for adequate communication was set at 3.17.

**Conclusions:**

Applying the protocol, the mean overall score rose above the level set in an earlier study for the MAAS-Global scores to represent ‘adequate GP communication behaviour’. Our findings indicate that incorporating context factors in communication assessment thus makes a meaningful difference and shows that context factors should be considered as ‘signal’ instead of ‘noise’ in GP communication assessment. Explicating context factors leads to a more deliberate and transparent rating of GP communication performance.

## Background

As communication is at the heart of good clinical practice, communication training and assessment are key components in undergraduate as well as postgraduate medical curricula. Communication levels are usually assessed by rating the performance against predefined communication skills [1-3]. Widely used communication assessment instruments such as the Maastricht History-taking and Advice Scoring list (MAAS-Global) [4], also applied in general practitioner’s (GP) performance assessment, determine to what extent generic communication skills, expected to be pursued in every consultation with every patient, are observed. However, generic criteria may fail to capture the contextual proficiency of the GP’s performance [2,5-7]. The underlying assumptions that generic communication skills should be applied in any consultation and that every consultation can be treated as if it requires the same communication performance seem unjustified. Several authors have argued that context factors influence communication in health care [1,8-10] and that communication performance is therefore context-dependent [11-16]. Moreover, from a patient-centred perspective, every consultation is unique and sets a specific context for the communication between the doctor and the patient [17-20].

So far, the role of the context in which doctor-patient communication takes place has hardly been accounted for in communication assessments [10,21,22]. However, the influence of context factors has been put forward as an explanation to why GPs achieve low scores on communication performance [23,24]. If context factors can be taken into account when rating GP communication skills, we may move from a generic to a context specific assessment of GP communication performance.

In a previous explorative study, several context factors (CF) were identified on the level of the consultation that may well explain deviations from generic recommendations on communication [25]. These context factors were related to the doctor, to the patient, and to the consultation. If, for example, the consultation has been initiated by the doctor to re-evaluate the patient’s condition, the doctor does not need to explore the patient’s request for help. Similarly, in case of an easily solved problem like ear wax blocking hearing, it seems spurious to explore emotions extensively. Yet some patients do fear the removal procedure, or may have questions concerning the consultation or treatment goal. Thus, context factors may explain why certain communication behaviour is absent but they do not justify its absence in all circumstances. This clearly reflects the dynamic way ‘context’ is to be understood [22]. By incorporating contextual influences, communication performance assessment may gain in validity.

The current exploratory study aimed to find out how communication performance ratings change if context factors are explicitly taken into account. Our research question was: How does incorporating context factors influence the assessment of GP communication performance? In order to answer this question, we first examined which previously identified context factors were present in the currently studied GP consultations. Secondly, we explored how applying a context-specific protocol would affect communication scores. We expected GP communication scores to be significantly higher if context factors are explicitly taken into account [25].

## Methods

### Sample selection

The study was carried out between February and September 2010. We selected a sample of 40 consultations from a database of 808 videotaped Dutch GP consultations, recorded as part of a video-observation study performed by NIVEL (Netherlands institute for health services research) in 2007 – 2008 [26]. The 40 GPs that participated in this NIVEL study have age, gender, practice type, and patients characteristics that are similar to the GP population characteristics in the Netherlands, although urban practices are over-represented [27]. Firstly, out of the 40 GPs, 20 GPs were randomly selected and subsequently, from each of these GPs, the 4th and 5th consultations were then selected, which we felt was a reasonable trade-off between analyzing GPs and consultations. We excluded the first three consultations during which the GP may have had to get used to the video recording. This procedure ensured sufficient power and variation between doctors and consultations. A sample of 40 consultations would provide enough power to establish a minimal relevant difference between the two ratings of 0.45 (α = 0.05, β = 0.10) on item scores [28]. The time interval between first (standard) and second (context-specific) rating was 5–6 months.

### Procedure

**Figure 1:**
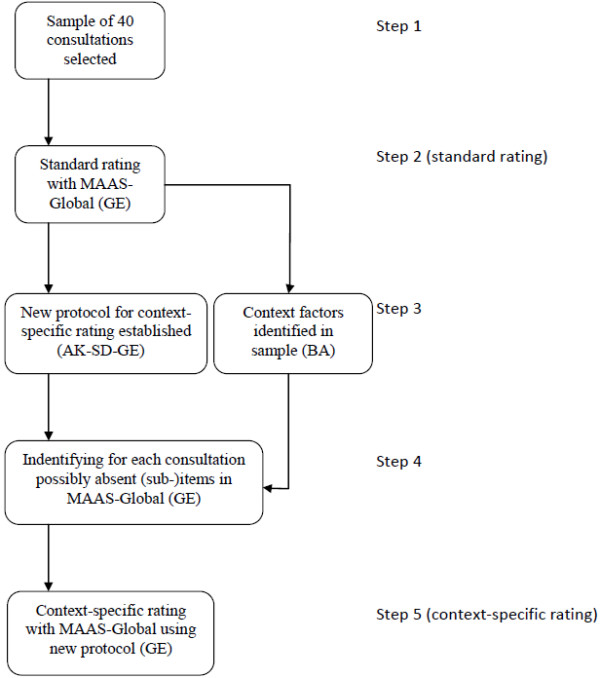
Procedure of incorporating context factors into communication assessment.

To answer the research question, one rater (GE), psychologist, communication trainer and assessor, rated the 40 consultations twice using the same rating instrument (the MAAS-Global) [29] (Figure 1). The first rating was performed in the standard way using the MAAS-Global Manual [30]. In the second, context-specific rating CFs were explicitly taken into account following the newly developed protocol. The MAAS-Global is a validated communication assessment instrument which serves as a guideline for patient-centred medical communication [2,4]. It is widely used in undergraduate medical and general practice specialty training programs in the Netherlands [31,32]. The MAAS-Global consists of 13 generic communication items that can be rated on a 7-point Likert scale, ranging from 0 (‘absent’) to 6 (‘excellent’). Two items can also be scored ‘Not applicable’. Each item has three or four sub-items that indicate criterion behaviour (Additional file 1). The MAAS-Global Manual offers guidelines to rate communication skills and acknowledges that CFs play a role, but leaves implicit how to incorporate contextual influences.

For the incorporation of CFs into the rating process, a context-specific rating protocol was developed by three researchers, each with different backgrounds (GP, communication researcher, communication trainer and assessor), to be used in addition to the MAAS-Global manual (Additional file 2). The protocol was developed on the basis of the relationships between CFs and the justifiable absence of communication skills found in our previous study [25]. It accounts for the absence of certain communication skills as a result of the presence of one or more CFs and at the same time keeps the existing rating rules of the MAAS-Global Manual intact. Additional file 2 presents the protocol and the ascertained relationships. In order to stay in line with the MAAS-Global rating rules, the central assumptions in the protocol were:

• If a sub-item is justifiably absent as a result of the presence of one or more CFs, it should not weigh on the item scores.

• If an item is justifiably absent due to the presence of one or more CFs, it should be scored ‘Not applicable’.

In between standard and context-specific rating, a second rater (BA), a GP and an experienced faculty member, rated the observed consultations for the presence of CFs, by using the list of previously identified CFs shown in Table 1 (Table 1 is presented in the Results section) [25] (see also Additional file 3 for examples how CFs were identified). Prior to the context-specific rating, GE received a data sheet from BA on the presence of CFs in each of the consultations. Differences on the identification of CFs that arose during the second rating were discussed between GE and BA until agreement was reached. Subsequently, GE noted the presence of the CFs on the MAAS-Global sheet for each consultation and indicated which of the new rating rules could be applicable in that consultation (see also in Results Table 2, second column). After this, the GP communication performance was rated the second time with the adapted MAAS-Global.

**Table 1:** List of context factors

**Doctor-related factors**		**Present in # consultations (%)**
1.	Doctor knows patient and his social context	29 (72)
2.	Doctor knows patients’ medical history	35 (88)
3.	Doctor knows patients’ way of communicating	29 (72)
4.	Doctor is very experienced	34 (85)
**Patient-related factors**		
5.	Specific patient **verbal behaviour**	11 (27)
6.	Specific patient **non-verbal behaviour**	1 (3)
7.	Patient is also treated by other provider	12 (30)
8.	Patient has a disease (diagnosis) or (recurrent) problem known to both doctor and patient	24 (60)
9.	Patient is familiar with (physical) examination (PE)	23 (58)
**Consultation-related factors**		
10.	Single consultation	23 (58)
11.	First consultation in a series^1^	4 (10)
12.	Follow-up consultation in a series^2^	11 (28)
13.	Consultation in a series based on protocol (initiative by doctor)^3^	1 (3)
14.	Consultation in preventive care (initiative doctor)^4^	1 (3)
15.	Diagnosed problem is easily solved	3 (8)
16.	Problem urgently needs medical care	1 (3)
17.	Diagnosed problem is mainly psychosocial	2 (5)
18.	There is more than one person (patient) present	12 (30)
19.	Characteristics of physical examination:	
	- simple	30 (75)
	- invasive	4 (10)
	- intimate	6 (15)
	- superficial	29 (73)
	- leaves room for talk	10 (25)
	- requires full attention	27 (68)

Context factors present in 40 GP consultations (+ frequency).

^1^a first consultation in a series is a consultation in which no diagnosis is being made and patient is referred for further tests, or in which doctor and patient agree to a follow-up consultation for check-up purposes.

^2^a follow-up consultation in a series is a second or consecutive consultation concerning a complaint or problem for which a referral was made, or that was agreed upon by patient and doctor. It is also marked as a follow-up consultation if the patient has been referred for specialist care (even if surgery took place) and the patient comes back to report on that. (no maximum time span is indicated)

^3^a protocol based consultation is a consultation, concerning a prior diagnosed complaint or problem, that is initiated by the doctor on the basis of a protocol or clinical practice guideline.

^4^a preventive consultation is a consultation that is initiated by the doctor on the basis of a protocol or clinical practice guideline (diagnosis or disease is not necessarily established).

**Table 2:** Results of application of context-specific rating protocol

**MAAS-Global item**	**N times item is influenced **^**(a)**^		**Score without CF**	**Score with CF**^**(b)**^	**Effect size**
	**Potentially**	**Actually**	**(SD)**	**(SD)**	
1. Introduction^†^	5	4	3.60 (0.93)	4.10 (0.90)	0.55
2. Follow-up consultation	29	29	3.83 (0.58)	4.42 (0.79)	0.87
3. Request for help^‡^	54	23	1.00 (1.45)	1.67 (2.23)	0.32
4. Physical Examination^‡^	83	31	4.33 (1.51)	5.14 (1.31)	0.57
5. Diagnosis^†^	76	6	3.84 (0.69)	4.46 (0.80)	0.80
6. Management	33	15	2.90 (1.15)	3.25 (1.15)	0.30
7. Closure^∞^	13	6	2.62 (0.92)	2.37 (2.19)	0.15
8. Exploration^‡^	72	18	1.85 (1.49)	2.57 (1.53)	0.48
9. Emotions^‡^	33	16	0.88 (1.27)	1.60 (1.94)	0.44
10. Information giving^†^	32	5	3.67 (0.97)	4.07 (1.05)	0.40
11. Summarizing^†^	0	0	3.27 (1.28)	4.00 (1.97)	0.42
12. Structuring^‡^	61	32	3.85 (1.21)	4.80 (1.36)	0.77
13. Empathy^‡^	0	0	4.25 (0.90)	4.87 (1.28)	0.56
Total mean score^‡^			2.98 (0.61)	3.66 (0.98)	0.84

^(a) ^Due to non-applicability of an item or a not-applicable sub-item. Items can be influenced by more than one sub-item that is not-applicable. If a (sub-)item is justifiably not applicable because of more than 1 context factor, it was counted only once.

^(b) ^Differences between scores without and with CF per item and for the total mean score were calculated with a paired samples t-test for equality of means.

^**∞**^ p < .05.

^**†**^ p < .01.

^**‡**^ p < .000.

### Data analysis

Kappa was calculated, based on a separate sample of seven consultations taken from the dataset, to assess inter-rater variance in determining the presence of CFs between the first (GE) and the second rater (BA) (κ = 0.69). Apart from checking the presence of the previously found CFs, their frequencies were calculated in order to determine to what extent applying the context-specific rating protocol could be expected to influence ratings of the MAAS-Global items.

The rating of the GP communication performance was done both times by the same rater (GE) to exclude noise produced by heterogeneity of raters [33]. To check for intra-rater consistency, kappa was calculated by twice scoring ten consultations that did not belong to the study sample, with a 6 month time lap between the two moments of assessment, using standard MAAS-Global rating rules (κ = 0.662).

To analyse the extent to which CFs influenced the rating process, we calculated the number of applicable MAAS-Global items per consultation, with and without accounting for CFs, as this number is used as the denominator to determine the overall score on the MAAS-Global [4,24,28,33]. Moreover, because context factors may predict the absence of certain communication behaviour (and thus a (sub-)item) [25] but do not necessarily lead to the absence of that specific behaviour, we calculated the number of times the MAAS-Global items were *potentially* influenced by CFs, based on applying the rules from the rating protocol (see Additional file 2), and compared this to the *actually* influenced number as a result of the ratings (see Table 2).

Our expectation that the mean item scores in the context-specific rating would be higher was tested by calculating the direction of the change in scores with a paired t-test for repeated measurement in the same sample, using PASW Statistics 18, Release Version 18.0.3 (SPSS, Inc., 2010, Chicago, IL, http://www.spss.com). To determine the relevance of the difference between the two ratings, the effect size was calculated for the difference between the individual MAAS-Global item scores and for the difference between the mean sum scores per consultation, divided by their pooled initial standard deviations (SDs); a *d* of 0.2 was considered a small effect, a *d* of 0.5 as a moderate effect, and a *d* of 0.8 as a large effect [33-35].

### Ethical approval

The study was performed according to Dutch privacy legislation. The privacy regulation was approved by the Dutch Data Protection Authority. All participating GPs and patients signed an informed consent form before the recording of the consultation. According to Dutch legislation, approval by a medical ethics committee was not required for this study.

## Results

The 20 GPs and the patients in the research sample were comparable in gender, age, and practice type to those of the larger data set (35% female GPs, mean age 49 yrs (SD: 6.4) vs. 51 yrs (SD: 5.9)) [26]. All context factors in the list were observed in the current sample, with frequencies varying from one time to 34 times. Table 1 presents the CFs observed in the consultations and their frequencies found in the study sample.

In 38 out of 40 consultations, CFs prompted the application of the context-specific rating protocol. In two consultations there was no CF present that required deviating from the MAAS-Global Manual. The mean number of CFs per consultation was 6.5 (range 4 – 12). As a consequence of incorporating context factors, the number of applicable items per consultation decreased from 12.2 to 11.8 (sub-items: from 40.6 to 37.7).

The potential change in scores was highest in eight out of thirteen items (items 3, 4, 5, 6, 8, 9, 10, and 12) whereas little to no change would be expected in five items (items 1, 2, 7, 11, and 13) (Table 2).

As a result of applying the context-specific rating protocol, a significant increase was found in ten out of the thirteen mean item scores whereas one item (item 7) showed a decrease (Table 2). In the items 2 (Follow-up consultation) and 6 (Management) the difference in scores was not significant. The mean overall score in the standard rating was 2.98, while in the context-specific rating it was 3.66 (p < 0.00). Effect sizes were large for three items and moderate for another four items. In the remaining six items effect sizes were low (Table 2). Effect size in the mean overall score was large (0.84).

## Discussion

This study indicates that explicitly incorporating context factors into communication assessment in a protocolized way leads to a significantly lower number of applicable MAAS-Global items per consultation, and to higher item scores. By applying the protocol, the mean overall score found in our study rose above the mean minimum standard score of 3.17, which is the level set for the MAAS-Global scores to represent ‘adequate GP communication behaviour’ in a study by Hobma [24]. However, consistent with other recent findings on doctor communication patterns [36-38], the GP scores in our study on the items Request for help, Management, Exploring and Emotions are below the minimum standard. These are important aspects in GP-patient communication and need attention in postgraduate GP training and continuing professional development (CPD).

In the standard rating protocol, the absence of criterion behaviour is penalized by a low item score, whereas judging a sub-item to be justifiably absent will lead to relatively higher item scores. In our study, most changes in item scores were as we expected them but the results in items 1, 6, 7, 11 and 13 were unforeseen. The unexpected results in these items may be due to rater leniency in the second rating, although the change did not go in the same direction in all items. The significant change in item 7 (Closure) can also be explained by the lack of clarity in the MAAS-Global protocol where the assessor has to score a question near the end of a consultation: either under Closure as ‘general question‘, or under Management (item 6) as ‘asking for patient’s response‘. An explanation for the not-significant change in item 6 (Management) may be that the potential change was not acknowledged in the actual rating: absence of sub-items was not justified or sub-items were not absent. Apparently, in our sample this item did not change under the influence of context factors as much as we expected, even if closing remarks were scored more often under Management the second round. However, for the results to be corroborated, a more robust study is necessary. As our study is exploratory, the numerical changes we found must definitely be interpreted with diligence.

The presence of contextual factors identified before is also confirmed, as all previously identified CFs in GP consultations were also found in the current sample [25]. However, CF frequencies found in this study cannot be generalized, as the sample is not sufficiently large. Although the representativity of GPs and patients in the sample is good, the frequencies only represent the consultations in the sample and were needed in this study to explore the magnitude of the effect on item scores. In our previous study, CFs were identified on the basis of inductive reasoning, using several rounds of systematic analysis to establish what factors could explain low scores.

Although there is wide recognition of the fact that professional competence is context-dependent, this aspect has so far been neglected in assessment of GP communication with patients in authentic consultations [6,7,10,11]. Now that we have found indications that, especially in workplace-based assessment, CFs can and need to be incorporated explicitly in judging communication performance, this way of assessment may enhance the credibility of communication training and assessment, not only for GPs but also in GP specialty training. Studies on GP trainee experiences [39,40] show that there is a need for this. The application of a context-specific protocol can do justice to clinical practice as it acknowledges the context-specificity of GP (trainee) communication in their surgeries. It may also contribute to removing the artifact that is created by merely looking at the presence of generic communication skills.

From recent research, we know that experts, when assessing trainee performance in practice, implicitly take contextual information into account [41-43]. By explicating CFs and by designing a context-specific assessment protocol, we may have unveiled part of the internal and implicit process of weighing contextual information. We have made this process explicit and thus open to empirical research. However, although the context-specific rating protocol was based on this study and developed by three researchers with different backgrounds, a limitation is that the protocol has not yet been reviewed by other GPs.

Explicitly accounting for CFs in workplace-based communication assessment can not only make performance scores more transparent, it may also raise their external validity. The characteristics of the various consultation-related CFs reflect current developments in family medicine in which a growing number of follow-up and preventive consultations concerning chronic disease management is seen for which protocols have been developed [44-46]. However, although the rating process using the context-specific protocol leads to a more refined outcome, it also encompasses a long list of items to ‘tick’. Adding extra criteria to the assessment process may render it less feasible in practice. Raters, however, need to be sensitized for context influences and do it justice in their assessments, and they can be trained to do so.

To determine the presence of context factors, some subjectivity is necessarily involved. As is shown in other studies on assessment of clinical performance, expert raters recognize context as an important factor modulating their assessment of, for instance, resident performance [41,43,47]. Although we chose optimal rater consistency by having the same rater for both the first and second rating of communication performance and substantial inter-rater agreement between two raters was found in determining the presence of CFs in GP consultations, the inherent limitation is that this may have caused a bias to corroborate the hypothesized findings, both in the first and in the second rating. Therefore, keeping in mind the exploratory nature of the study, the results should be interpreted with care.

More research is needed to validate the currently established context factors and the protocol that we developed more rigorously. Moreover, as contexts change with time, also the way it will influence GP communication in their patient encounters will change. Therefore, context factors as well as a context-specific rating protocol will need to be updated regularly.

## Conclusion

Assessment of professional performance is a complex enterprise, as so many behaviours seem to depend on contextual factors [22]. We think that context factors should rather be considered as ‘signals’ than as ‘noise’ in GP communication assessment and that the results of our study in incorporating them are promising. Now, a more robust study can be carried out to find out if our results are generalisable. The context-specific rating protocol should be reviewed by other GPs, and other raters should apply the protocol to assess GP consultations to further validate our findings. Furthermore, for validation of the protocol, research should focus on experts rating communication and the way they incorporate contextual information in assessing communication performance.

Although we do not claim to have found all relevant context factors in GP communication, the presence of CFs we did find and their influences on GP communication plead for a more context-specific approach of communication assessment, as has been advocated before [7,10]. Evidently, communication competence “is not defined solely by the presence or absence of specific behaviour, but rather by the presence and timing of effective verbal and non-verbal behaviour within the context of individual interactions with patients or families” [2].

## Competing interest

The authors declare that they have no competing interest.

## Authors’ contributions

GE carried out the communication performance rating and drafted the manuscript. BA carried out the assessment of CFs. GE, AK and SD conceived of the study and participated in its design and coordination. GE performed the statistical analysis. All authors read, contributed to and approved the final manuscript.

## Pre-publication history

The pre-publication history for this paper can be accessed here:

http://www.biomedcentral.com/1471-2296/14/65/prepub

## Supplementary Material

Additional file 1 MAAS-Global rating list for doctor-patient communication skills. Click here for file

Additional file 2 Context-specific rating protocol. Click here for file

Additional file 3 **Examples of how context factors for GP – patient encounters were identified **[25]**.** Click here for file

## Declaration

Funding: The study was funded by the SBOH Foundation, employer of Dutch Family Medicine Residents and funder of the national residency program for GPs in the Netherlands.
