# Supplementary material for: Context factors in general practitioner - patient encounters and their impact on assessing communication skills - an exploratory study
Source: BMC Fam Pract. 2013 May 22;14:65. doi: 10.1186/1471-2296-14-65 (PMC3688246; doi:10.1186/1471-2296-14-65)
Supplement: Additional file 1 — MAAS-Global rating list for doctor-patient communication skills. [file 1471-2296-14-65-S1.pdf]

Appendix 1

MAAS-Global rating list for doctor-patient communication skills.

Each item is scored on a scale ranging of 0-6. Under the new rating rules, for the items 2, 4, 5 and 9 the rating 'not applicable' is an additional option.

Communication skills for each separate phase.	General communication skills
**1. Introduction**<br>• giving the patient room to tell his story<br>• general orientation on the reason for visit<br>• asking about other reasons for visit<br><br>**2. Follow-up consultation**<br>• naming previous complaints, requests for help and management plan<br>• asking about adherence to management plan<br>• asking about the course of the complaint<br><br>**3. Request for help**<br>• naming requests for help, wishes or expectations<br>• naming reasons that prompted the patient to come now<br>• completing exploring request for help<br><br>**4. Physical examination**<br>• instructions to the patient<br>• explanation of what is being done<br>• treating the patient with care and respect<br><br>**5. Diagnosis**<br>• naming findings and diagnosis ⁄ hypothesis<br>• naming causes or the relation between findings and diagnosis<br>• naming prognosis or expected course<br>• asking for the patient's response<br><br>**6. Management**<br>• shared decision making, discussing alternatives, risks and benefits<br>• discussing feasibility and adherence<br>• determining who will do what and when<br>• asking for patient's response<br><br>**7. Evaluation of consultation**<br>• general question<br>• responding to requests for help<br>• perspective for the time being	**8. Exploration**<br>• exploring requests for help, wishes or expectations<br>• exploring patient's response to information given within patient's frame of reference<br>• responding to non-verbal behaviour and cues<br><br>**9. Emotions**<br>• asking about ⁄ exploring feelings<br>• reflecting feelings (including nature and intensity)<br>• sufficiently throughout the entire consultation<br><br>**10. Information giving**<br>• announcing, categorizing<br>• in small quantities, concrete explanations<br>• understandable language<br>• asking whether the patient understands<br><br>**11. Summarizations**<br>• content is correct, complete<br>• concise, rephrased<br>• checking<br>• sufficiently throughout the entire consultation<br><br>**12. Structuring**<br>• logical sequence of phases<br>• balanced division of time<br>• announcing (history taking, examination, other phases)<br><br>**13. Empathy**<br>• concerned, inviting and sincerely empathetic in intonation, gesture and eye contact<br>• expressing empathy in brief verbal responses
