# Supplementary material for: Context factors in general practitioner - patient encounters and their impact on assessing communication skills - an exploratory study
Source: BMC Fam Pract. 2013 May 22;14:65. doi: 10.1186/1471-2296-14-65 (PMC3688246; doi:10.1186/1471-2296-14-65)
Supplement: Additional file 2 — Context-specific rating protocol. [file 1471-2296-14-65-S2.pdf]

## Appendix 2 Context-specific rating protocol (summary)

Specific rating rules per context factor

| | If this context factor is present | then this (sub)item can justifiably be absent* | |
|---|---|---|---|
| | **doctor-related factors** | | |
| 1. | doctor knows patient and his social context | 6.1 | shared decision making |
| | | 8.3 | explores within patient's frame of reference |
| | | 10.3 | uses understandable language |
| 2. | doctor knows patients' medical history | 5.3 | names findings and hypothesis / diagnosis |
| | | 5 | Diagnosis |
| | | 8.3 | explores within patient's frame of reference |
| 3. | doctor knows patients' way of communicating | 6.1 | shared decision making |
| | | 8.3 | explores within patient's frame of reference |
| | | 8.4 | responds to non-verbal behaviour and cues |
| | | 9.1 | explores / discusses feelings |
| | | 9.2 | reflects feelings |
| 4. | doctor is very experienced | 12.1 | logical sequence of phases |
| | | 12.2 | balanced division of time |
| | **patient-related factors** | | |
| 5. | specific patient verbal behaviour | 3.1 | names request for help or expectations |
| | | 3.2 | names reason for attending |
| | | 7.2 | checks response to request for help |
| | | 8.1 | explores request for help or expectations |
| 6. | specific patient non-verbal behaviour | - | |
| 7. | patient is also treated by other provider | 5.1 | names findings and hypothesis / diagnosis |
| | | 12.2 | balanced division of time |
| 8. | patient has a disease (diagnosis) or (recurrent) problem known to both doctor and patient | 4 | Physical Examination |
| | | 5.1 | names findings and hypothesis / diagnosis |
| 9. | patient is familiar with (physical) examination (PE) | 4.1 | gives instructions to patient |
| | | 4.2 | explains what is done in PE |
| | **consultation-related factors** | | |
| 10. | single consultation | 2 | Follow-up consultation |
| 11. | first consultation in a series | 2 | Follow-up consultation |
| 12. | follow-up consultation in a series | 3.1 | names request for help or expectations |
| | | 3.2 | names reason for attending |
| | | 4 | Physical Examination |
| | | 5.1 | names findings and hypothesis / diagnosis |
| | | 12.2 | balanced division of time |
| 13. | consultation in a series based on protocol (initiative by doctor) | 3.1 | names request for help or expectations |
| | | 3.3 | completes exploration request for help |
| | | 4 | Physical Examination |
| | | 5.2 | names cause or relationship between findings and diagnosis |
| | | 7.2 | checks response to request for help |
| | | 8.1 | explores request for help or expectations |
| 14. | consultation in preventive care (initiative doctor) | 1.2 | general orientation on reason for visit |
| | | 1.3 | asking about other reason for visit |
| | | 2 | Follow-up consultation |
| | | 3.1 | names request for help or expectations |
| | | 3.3 | completes exploration request for help |
| | | 4 | Physical Examination |
| | | 5 | Diagnosis |
| | | 7.2 | checks response to request for help |
| | | 8.1 | explores request for help or expectations |
| | | 9 | Emotions |
| | | 12.1 | logical sequence of phases |
| 15. | diagnosed problem is easily solved | 9.1 | explores / discusses feelings |
| | | 9.2 | reflects feelings |
| 16. | problem urgently needs medical care | 1.3 | asking about other reason for visit |
| | | 3.1 | names request for help or expectations |
| | | 6.1 | shared decision making |

| | | | |
|---|---|---|---|
| | | 8.1 | explores request for help or expectations |
| | | 12.2 | balanced division of time |
| 17. | diagnosed problem is mainly psychosocial | 12.1 | logical sequence of phases |
| | | 12.2 | balanced division of time |
| 18. | there is more than one person (patient) present | - | |
| 19. | characteristics of physical examination | 4.1 | gives instructions to patient |
| | | 4.2 | explains what is done in PE |

* Numbers coincide with (sub)items on MAAS-Global (see Appendix 1)
