# Supplementary material for: Context factors in general practitioner - patient encounters and their impact on assessing communication skills - an exploratory study
Source: BMC Fam Pract. 2013 May 22;14:65. doi: 10.1186/1471-2296-14-65 (PMC3688246; doi:10.1186/1471-2296-14-65)
Supplement: Additional file 3 — Examples of how context factors for GP – patient encounters were identified [25]. [file 1471-2296-14-65-S3.docx]

**Additional file 3: Box 1 Examples of how context factors for GP – patient encounters were identified [**[**25**](#_ENREF_25)**]**

In our previous study we used an inductive reasoning approach to identify context factors, acting on a micro-level of the GP – patient encounter, that could explain low scores in communication performance.

For example, we observed the patient and GP discussing the patient’s social and/or family circumstances (e.g. a patient who had recently had to move to a smaller house; a patient with a partner who has a serious health condition), or referring to prior contacts (e.g. in a consultation with a child that was taciturn and very difficult to engage) in the beginning of the encounter. In these encounters, the GP did not explore the patient’s context. From this, we inferred that there was a doctor-related context factor ‘knows the patient and his/her social context’ and ‘knows the patient’s way of communicating’, that could explain a low score on ‘Exploration’.

We also observed patients at the beginning of the consultation who, unsolicited, stated clearly and in detail their health problem and related needs, preferences and expectations. The GP’s response in these cases was restricted to a few additional clarifications or a very short history taking prior to proceeding to the physical examination. We also observed a patient who persevered in asking questions - out of anxiety or as a security check. This seemed to affect the GP’s communication,

leading to a focus on answering the questions and providing reassurance, but also to a decrease in expressed empathy. From these observations, we identified the patient-related context factor ‘specific verbal behavior’, which, in the first case, could also explain a low score on ‘Exploration’ and, in the second case, could explain a low score on ‘Empathy’.

Furthermore, we observed a difference between follow-up and preventive consultations – initiated by the GP – on the one hand, and on the other hand single consultations, first consultations in a series, and other follow-up consultations, in which the patient presented with a problem and the initiative to attend mainly lay with the patient. If, for instance, the initiative for a consultation lies with the GP, this would explain a low score on ‘Exploring the patient’s request for help’. Obviously, if asked for the reason for the encounter, the patient would reply: “I’m here because you asked me to be”. These differences were identified as consultation-related context factors.

Also specific aspects of the presented problem were inferred as consultation-related factors. For example, in dealing with complaints that were easily solved (e.g. removing cerumen or treating chicken-pox), we saw the GP not going into emotions. We inferred that, as these complaints usually have little emotional impact, there is no need for the GP to discuss emotions. Thus, this context factor could explain a low score on ‘Emotions’.
